# Unfolding the Human Milk Microbiome Landscape in the Omics Era

**DOI:** 10.3389/fmicb.2019.01378

**Published:** 2019-06-25

**Authors:** Lorena Ruiz, Cristina García-Carral, Juan Miguel Rodriguez

**Affiliations:** ^1^Department of Microbiology and Biochemistry of Dairy Products, Instituto de Productos Lácteos de Asturias (IPLA-CSIC), Villaviciosa, Spain; ^2^Department of Nutrition and Food Science, Complutense University of Madrid, Madrid, Spain

**Keywords:** microbiome, human milk, breastfeeding, infant nutrition, microbiome modulation

## Abstract

Studies conducted in the last years have demonstrated that human milk represents a continuous supply of beneficial bacteria to the infant gut, which contribute to the maturation of the digestive and immune functions in the developing infant. Nevertheless, the origin of bacterial populations in milk is not fully understood yet and they have been proposed to originate from maternal skin, infant’s mouth, and (or) endogenously, from the maternal digestive tract through a mechanism involving immune cells. Understanding the composition, functions and assembly of the human milk microbiota has important implications not only for the infant gut microbiota establishment, but also for the mammary health since dysbiosis in the milk bacteria may lead to mastitis. Besides, host, microbial, medical and environmental factors may affect the composition of the human milk microbiome, with implications for the mother-infant health. Application of both culture-dependent and -independent techniques to assess the milk microbiome faces some practical limitations but, together, have allowed providing novel and complementary views on its origin, composition and functioning as summarized in this minireview. In the next future, the application of the ultimate advances in next-generation sequencing and omics approaches, including culturomics, will allow a detailed and comprehensive understanding of the composition and functions of these microbial communities, including their interactions with other milk components, expanding the opportunities to design novel microbiome-based modulation strategies for this ecosystem.

## Human Milk Contains a Microbiome

Human milk is the gold standard for infant nutrition as it contains multiple biologically active components, including immune factors, lipids, oligosaccharides, microRNAs and hormones among others (Hennet and Borsig, 2016), whose concentration vary with maternal and environmental factors, getting adapted to the variable needs of the baby (Hunt et al., 2011; Andreas et al., 2015; Feng et al., 2016; Gomez-Gallego et al., 2016; Kumar et al., 2016; Ruiz et al., 2017). Globally, its complex and dynamic composition promotes a healthy infant growth and development, which has prompted international organizations to recommend exclusive breastfeeding during the first 6 months of life (World Health Organization, 2003).

Microorganisms have emerged as important bioactive components of human milk. Its occurrence in milk was first recognized the second half of the past century, while studying the potential transmission of infections through breastfeeding (Rantasalo and Kauppinen, 1959; Foster and Harris, 1960; Kenny, 1977; Eidelman and Szilagyi, 1979). Later, several studies described the presence of viable commensal, mutualistic or potentially probiotic bacteria in healthy human milk (Fernández et al., 2013), leading to an increasing interest in human milk microbiota and their effects in maternal-infant health. Indeed, among the microorganisms occurring in milk, some strains belonging to the species *Lactobacillus salivarius, Lactobacillus fermentum, Lactobacillus gasseri, Bifidobacterium breve, Bifidobacterium adolescentis*, and *Bifidobacterium longum* subsp. *infantis* have demonstrated potential to promote mother and infant health, including the prevention or treatment of lactational mastitis, the promotion of a normal gut bacterial colonization in preterm neonates, or the amelioration of diarrhea in IBS patients (Arroyo et al., 2010; Chiu et al., 2014; Maldonado-Lobón et al., 2015; Moles et al., 2015; Fernández et al., 2016; Shin et al., 2018).

The scientific interest in human milk bacteria is evolving in parallel to the unveiled roles of human microbiomes in health and disease, and to the advances in the methodologies available for their study. Nowadays, research interests in this field are focused on (a) the roles that milk bacterial communities play on infant health and development, including their influence on the acquisition of the gut microbiota in early life; (b) the roles that they may play on maternal health, including breast health; (c) the maternal, infant, environmental and medical factors that shape and modulate their compositions, both under physiological conditions and when dysbiosis occurs; and (d) their origin, in order to confirm or refuse the existence of an endogenous oral-entero-mammary route allowing the selective translocation of some bacteria from the maternal digestive tract to the mammary gland.

The ever-growing asset of-omic tools is opening new avenues to understand the benefits of breastfeeding from microbiological, metabolic and immunological perspectives, and to establish new microbiota-based strategies to promote maternal-infant health, including the development of procedures for the transfer of the milk microbiota to neonates or infants that are not breastfed or the development of human milk-derived probiotics (Fernández et al., 2018). This review summarizes the main advances on our comprehension of the human milk microbiota gained through the refinement of tools available for microbial ecology studies, identifying opportunities to address existing knowledge gaps.

## Culture-Dependent Based Studies

Microbiology studies have traditionally relied on culturing, isolating and characterizing, phenotypically and (or) genotypically, the bacterial isolates, though these approaches fail to obtain information on non-culturable bacteria. Additionally, most microbial studies on milk were performed on samples from women whose infants were suffering from potential milk-borne infections and thus, microbial growth media and conditions have been biased toward detecting pathogens including viral particles (cytomegalovirus, Zika, HIV, etc.), and pathogenic microbial species typically associated with mammary infections (e.g., *Staphylococcus aureus*) or infant infections (e.g., group B *Streptococcus*) (Chen et al., 2016). Overall, culturing conditions for such pathogenic bacteria require the utilization of standard clinical cultivation media and relatively short incubation periods (16–18 h) at 37°C under standard atmospheric incubations. These conditions have allowed isolating dominant and rapidly growing culturable microorganisms mainly including Gram-positive species (*Staphylococcus, Streptococcus, Corynebacterium*, and *Propionibacterium*). Other bacterial groups present in milk from healthy lactating mothers are more fastidious to grow under routine laboratory conditions as they require either specific atmospheric incubations (e.g., anaerobic conditions) or specific nutrients or ingredients in the cultivation media (for instance, reducing agents for growing anaerobic species), and thus may have passed unnoticed in early microbiological studies not specifically designed to recover such species. For instance, lactic acid bacteria (*Lactobacillus, Lactococcus, Leuconostoc, Weissella*...) and bifidobacteria have only been isolated from milk following the utilization of specific growth media and longer anaerobic incubations (Martín et al., 2003, 2009; Abrahamsson et al., 2009; Solís et al., 2010; Arboleya et al., 2011; Murphy et al., 2017). Globally, over 200 different bacterial species representing approximately 50 different genera have been isolated from milk, including new bacterial species, such as *Streptococcus lactarius* (Martín et al., 2011).

Culture-dependent approaches have been pioneering in demonstrating that healthy human milk harbors a microbial community, representing a source of commensal microorganisms for the neonate. Albeit their limitations to access unculturable bacteria, culturing enables preserving the strains facilitating further studies and exploitation of potential biotechnological applications (Lara-Villoslada et al., 2007; Arboleya et al., 2011; Langa et al., 2012; Cárdenas et al., 2014, 2015). Specifically, the activities most commonly studied on milk isolates include virulence and antibiotic resistance (Jiménez et al., 2008a; Kozak et al., 2015), but also bacteriocin production (Heikkilä and Saris, 2003; Olivares et al., 2006; Lara-Villoslada et al., 2007; Kozak et al., 2015; Sharma et al., 2017) and probiotic traits (Fernández et al., 2013; Reis et al., 2016). Indeed, bacteria from milk of healthy women fulfill the criteria recommended for human probiotics: human origin, a history of safe prolonged intake, adaptation to mucosal and dairy substrates (Lara-Villoslada et al., 2007; Fernández et al., 2013), and to the human gut (Jeurink et al., 2013). Thus, some *Lactobacillus* and *Bifidobacterium* species (*L. gasseri, L. salivarius, L. rhamnosus, L. plantarum, L. fermentum, L. reuteri, B. breve, B. longum*…) from milk have awakened strong interest as potential probiotic bacteria, enjoying the GRAS (Generally Recognized As Safe) and the QPS (Qualified Presumption of Safety) status conceded by the Food and Drug Administration and the European Food Safety Authority, respectively.

Bacterial isolation coupled to phenotypical and genotypical characterization, including whole genome sequencing (WGS) analyses, are essential steps to characterize and evaluate the safety and the probiotic potential of bacterial isolates from human origin, including those isolated from milk. Generally, the *in vitro* assessment of probiotic properties in human milk isolates has included their antimicrobial activity against pathogenic microorganisms and the compounds that may be responsible for such activity (organic acids, bacteriocins, hydrogen peroxide), the ability to survive when exposed to conditions similar to those found in the human gastrointestinal tract, their adherence to human intestinal cells and mucin, their ability to degrade mucin or to produce biogenic amines or their susceptibility to antibiotics (Martín et al., 2005, 2006, 2010). The safety and health-promoting properties of some strains have also been confirmed *in vivo*, including the use of animal models (Olivares et al., 2006) and human clinical trials (Jiménez et al., 2008b; Arroyo et al., 2010; Maldonado et al., 2010, 2012; Gil-Campos et al., 2012; Espinosa-Martos et al., 2016; Fernández et al., 2016). Finally, a few strains isolated from this fluid have been analyzed to date through WGS, including representatives from the genera *Lactobacillus, Bifidobacterium*, and *Streptococcus* (Jiménez et al., 2010a,b, 2012; Martín et al., 2012, 2013).

In the framework of recent research conducted in the field of the gut microbiota, the “culturomics” approach, which is based on the utilization of a wide range of culturing and incubation conditions, is emerging as a promising strategy to aid in the discovery and description of novel microorganisms associated to the human host, including pathogenic and commensal species (Bilen et al., 2018). Such approach has not been applied to the human milk ecosystem yet, although it can be anticipated that it will lead to significant advances in our knowledge of this human ecosystem and to the discovery of novel health-promoting bacterial strains.

## Culture-Independent Based Studies

Since cultivable microorganisms represent a fraction of the communities inhabiting a specific ecological niche, culture-independent molecular techniques, i.e., quantitative PCR, denaturing (or temperature) gradient gel electrophoresis, and Next generation sequencing (NGS) approaches, from metataxonomics (16SrRNA amplicon analysis) to metagenomics (total DNA sequencing), have allowed valuable complementary assessment of the human milk microbiota, including information on microorganisms which remain unculturable to date (Jeurink et al., 2013; McGuire and McGuire, 2015; Mira and Rodriguez, 2017). Nonetheless, such techniques introduce other limitations and biases as they may over- or underestimate some groups because of differences in cell structures affecting DNA extraction efficiencies (McGuire and McGuire, 2015; Gomez-Gallego et al., 2016; Mira and Rodriguez, 2017). For instance, it has been well established that differences in the storage and manipulation of the samples and in the procedures for DNA extraction and storage strongly affect the yield and quality of extracted DNA, but may also lead to the underrepresentation of those microorganisms more recalcitrant to cellular lysis (Gram-positive bacteria or eukaryotic species), strongly biasing the results obtained from microbiome analysis of complex microbial communities (Wesolowska-Andersen et al., 2014), and precluding the comparison of results from different studies. While strong efforts have attempted to standardize methodologies so as to allow comparison of results from different studies in the frame of the human gut microbiome, such standardization has not been implemented in human milk microbiome studies, yet.

Besides, culture-independent techniques cannot differentiate between live and dead microorganisms, as DNA from dead organisms may persist in the environment, affecting the biological significance of the conclusions from sequencing-based analysis of microbial communities. Several methodologies have been implemented to incorporate a viability assessment in DNA-based analysis (Emerson et al., 2017). Among them, the use of propidium monoazide (PMA) should be highlighted. PMA is a dye which penetrates damaged membranes and, thus, only binds to extracellular DNA or DNA from damaged cells. Upon light exposure, PMA irreparably damage the DNA and, consequently, further DNA extraction and amplification procedures will only affect live cells. Nevertheless, such methodologies have not been widely implemented in microbiome-based surveys because of their current drawbacks; as an example, their efficacy to differentiate live and dead cells may depend on the particular composition of the community (Li R. et al., 2017). Finally, other limitations are inherent to the specific 16S rRNA region(s) targeted, sequencing platform used and/or the specific bioinformatic analysis, particularly in terms of the criteria followed to filter sequencing errors, sequencing clustering methods, and reference databases employed for taxonomic assignation or functional annotation (Fouhy et al., 2016). These facts highlight the urgent need to standardize methodologies for the sequencing-based assessment of microbial populations within the human body, including specific procedures adapted to the particular characteristics of different human body ecosystems (e.g., samples with a low biomass or with a high proportion of Gram-positive bacteria, such as human milk).

Anyhow, culture-independent studies confirmed the presence in milk of DNA from bacterial groups previously isolated from this ecosystem, including *Staphylococcus, Streptococcus, Corynebacterium, Propionibacterium*, lactic acid bacteria, and *Bifidobacterium* (Martín et al., 2007; Collado et al., 2009; Hunt et al., 2011; Cabrera-Rubio et al., 2012, 2016; Jost et al., 2013, 2014; Ward et al., 2013; Jiménez et al., 2015; Fitzstevens et al., 2017); but also reported the presence of DNA from microbial groups which had not been previously recovered through culturing healthy human milk samples, such as *Clostridium* and *Bacteroides* (Delgado et al., 2008; Collado et al., 2009). Overall, these techniques offered novel tools to study the milk ecosystem and defined some changes in total or specific microbial groups representation and in microbial activities of interest, e.g., virulence or bacteriocin production (Obermajer et al., 2015) linked to specific maternal/infant or environmental characteristics (Table 1). For instance, qPCR-based studies associated antibiotic administration to reduced lactobacilli and bifidobacterial presence (Soto et al., 2014) and identified reduction in bifidobacterial presence in association to preterm delivery-(Khodayar-Pardo et al., 2014).

**Table 1:** Summary including the main conclusions and experimental design of culture-independent studies reported to date on human milk microbiota.

Study group	Methodology	Factor study	Main conclusions	References
Healthy women (Spain), (*n* = 65)	qPCR and 28S rRNA sequencing	–	Detection of fungi species in a high proportion of samples from healthy lactating women	Boix-Amorós et al., 2017
Healthy women (Spain), (*n* = 32)	qPCR using 16S rRNA for target populations	Preterm vs. Term deliveries Longitudinal study	Prematurity was associated with reduced *Bifidobacterium* levels C-section was associated with reduced *Streptococcus* in transitional milk	Khodayar-Pardo et al., 2014
Healthy women (Germany and Austria) (*n* = 160)	qPCR using 16S rRNA for target populations	Antibiotherapy effect	Reduced lactobacilli or bifidobacteria positive samples following antibiotherapy.	Soto et al., 2014
Healthy women (United States), Longitudinal study (*n* = 16)	16S rRNA sequencing (V1–V2 regions/454 platform)	Longitudinal study in healthy lactating women	High inter-individual variability. Define a 9 core microbial genera including: *Staphylococcus, Streptococcus, Serratia, Pseudomonas, Corynebacterium, Ralstonia, Propionibacterium, Sphingomonas, Bradyrhizobiaceae*	Hunt et al., 2011
Healthy women (Finland), Longitudinal study (*n* = 18)	16S rRNA sequencing (V3–V4 regions/454 platform)	Longitudinal study and Maternal BMI	Mature milk contains a higher representation of oral inhabitants Maternal BMI and weight gain during pregnancy positively associated with staphylococci and lactobacilli; but negatively associated with bifidobacteria Elective C-section impacts the milk microbiota structure	Cabrera-Rubio et al., 2012
Healthy women (Spain), (*n* = 10)	16S rRNA sequencing (V1–V3 regions/454 platform)	Delivery mode	C-section is associated with higher abundance of *Staphylococcus* and lower of *Streptococcus*; and lower overall diversity	Cabrera-Rubio et al., 2016
Healthy women (Canada), (*n* = 39),	16S rRNA sequencing (V6 regions/Illumina Miseq platform)	Term vs. preterm, vaginal vs. C-section	High inter-individual variation, though gestation, mode of delivery or infant gender were not associated with specific milk microbiota differences	Urbaniak et al., 2016
Healthy women (Mexico) (*n* = 10)	16S rRNA sequencing (V4 regions/Illumina Hiseq platform)	Pre-pregnancy BMI, Comparison to infant saliva microbiota	Dominant genera display statistically significant differences in their representation in breast milk and infant saliva Higher abundance and diversity of *Streptococcus* than in other studies Pre-pregnancy BMI correlated with lower *Streptococcus* abundance and higher microbial diversity	Davé et al., 2016
Healthy women (Spain), (*n* = 21)	16S rRNA sequencing (V1–V4 regions/454 platform)	Longitudinal study from colostrum to mature milk	Define a core of 7 genera: *Finegoldia, Streptococcus, Corynebacterium, Staphylococcus, Acinetobacter, Peptoniphilus*, and *Pseudomonas* No differences in the representation of the most abundant genera at different time points	Boix-Amorós et al., 2016
Healthy lactating women (*n* = 8) vs. women receiving chemotherapy (*n* = 1)	16S rRNA sequencing (V6 region/Ion Torrent platform) + metabolomics	Chemotherapy	Chemotherapy associated with decreased bacterial diversity in human milk including shifts in *Bifidobacterium, Eubacterium, Staphylococcus, Cloacibacterium, Acinetobacter, Xanthomonadaceae*, and *Stenotrophomonas* Docosahexaenoic acid and inositol were also reduced	Urbaniak et al., 2014b
Healthy women (*n* = 79) (China, South Africa, Finland, and Spain)	16S rRNA sequencing (V4 region/Illumina Miseq platform) + metabolomics	Geographical and delivery type-associated variation	Variation in microbial and metabolic profiles, geographically and in association with type of delivery Proteobacteria, Actinobacteria and Bacilli show statistical significant correlations with some of the milk metabolites evaluated, including human milk oligosaccharides, lactate, polyamines and riboflavin	Gómez-Gallego et al., 2018
Same cohort as in Gómez-Gallego et al., 2018	16S rRNA sequencing (V4 region/Illumina Miseq platform) + metabolomics	Geographical variation	Geographical variation in breastmilk microbiota composition, and association with fatty acid profiles Expand previous universal core microbiome definitions, including 23 groups, and taxa specifically associated with certain geographical locations	Kumar et al., 2016
Healthy women (*n* = 41) (Centro African Republic)	16S rRNA sequencing (V1–V3 region/Illumina Miseq platform)	Lifestyle variation: hunter-gatherers vs. horticulturalists	Most abundant taxa very different from those reported in cohorts, including groups not previously detected in milk e.g., *Rhizobium* Seasonal and lifestyle differences related to variation in breastmilk microbiome composition, likely associated with variation in environmental and dietary exposures	Meehan et al., 2018
Healthy women (*n* = 33) (Taiwan)	16S rRNA sequencing (V3–V4 region/Illumina Miseq platform)	Longitudinal study	Describes the interindividual variability in the prevalence and relative abundance of commensal and opportunistic pathogenic bacteria in breastmilk from healthy women	Chen et al., 2018
Healthy women (*n* = 29), (Italy)	16S rRNA sequencing (V2 and V3 region/Ion Torrent platform)	Delivery type	Define variations in colostrum based on delivery type, with an overall greater abundance of environmental bacteria in samples from mothers that underwent C-section	Toscano et al., 2017
Healthy lactating women (*n* = 10) vs. mastitis (*n* = 10), (Spain)	Shotgun sequencing (454 platform)	Mastitis	Detect the presence or archaea, viruses, fungi and protozoa Mastitis relates to reduced microbiota diversity	Jiménez et al., 2015
Healthy women (*n* = 10) (Canada)	Shotgun metagenomics (Illumina platform)	na	Identify functional differences in breastmilk microbiome as compared to the gut microbiome of formula fed and breast fed infants Identify presumptive immunomodulatory DNA motifs	Ward et al., 2013
Healthy women (*n* = 18) vs. women with mastitis (*n* = 32)	16S rRNA sequencing (V2–V3 region/Ion Torrent platform)	Mastitis	Milk samples of mastitis women have lower microbial diversity, increased abundance of opportunistic pathogens and depletion of commensal obligate anaerobes.	Patel et al., 2017
Healthy women (*n* = 393) (Canada)	16S rRNA sequencing (V4 region/Illumina MiSeq platform)	na	Breastfeeding practices, maternal BMI and parity are significantly associated with milk microbiota coposition	Moossavi et al., 2019
Healthy women (*n* = 394) (Ethiopia, Gambia, United States, Ghana, Kenya, Peru, Spain, Sweden)	16S rRNA sequencing (V1–V3 region/Illumina Miseq platform)	Different geographical settings and relationship to infant fecal microbiotas	Geographical variation in total and core breastmilk microbiota, and a high percentage of taxa (70–88%) are encountered in both breast milk and infant fecal samples Positive correlations between the milk microbiota and the infant fecal microbiota	Lackey et al., 2019

## High-Throughput Culture-Independent Based Studies

The description of commensal bacteria in milk was coincidental in time with the advent of NGS approaches, which permit a deeper resolution of microbial communities and revealed that the complexity of milk microbiota is much more extensive than previously anticipated (Jeurink et al., 2013; McGuire and McGuire, 2017). Most NGS-based studies conducted on milk have focused on metataxonomics to define bacterial populations presence and their variation with selected factors (Table 1). Briefly, such studies revealed the existence of large inter-individual variations (Hunt et al., 2011; Cabrera-Rubio et al., 2012), some of which have been associated with geographical and lifestyles differences, with a general higher microbial diversity in samples from developing and (or) rural locations (Kumar et al., 2016; Sakwinska et al., 2016; Drago et al., 2017; Li S.-W. et al., 2017; Lackey et al., 2019), in agreement with observations within the gut microbiota ecosystem. Other specific factors frequently associated with overall variation in the milk microbiome structure include delivery mode (Cabrera-Rubio et al., 2012, 2016; Khodayar-Pardo et al., 2014; Toscano et al., 2017) and time from birth (Cabrera-Rubio et al., 2012, 2016; Khodayar-Pardo et al., 2014; Toscano et al., 2017 (Ma et al., 2015; Yu et al., 2016; Patel et al., 2017), though some inconsistencies have been reported among studies. For instance, differences in the milk microbiome structure of women who gave birth vaginally and those delivering by C-section have been frequently reported, though the specific taxa driving such differences seem to be study- and cohort-dependent: a higher abundance of lactobacilli species has been associated with C-section in South African, Finnish, Chinese and Taiwanese cohorts, whereas such difference has not been detected in Spanish women (Khodayar-Pardo et al., 2014; Kumar et al., 2016), some authors have even reported a higher lactobacilli representation following vaginal delivery in a Taiwanese population (Chen et al., 2018), and other studies found no differences in milk microbiomes depending on delivery type (Sakwinska et al., 2016; Urbaniak et al., 2016). Remarkably, particular cohort-dependent factors that might be driving variation in the human milk microbiomes have not been thoroughly explored, but might contribute to explain these apparent discordances. Similarly, differences between colostrum, transition and mature milk microbiotas have been reported by some authors, who reported an increased abundance of typical oral inhabitants in transition and mature milk, though such differences have not been consistently observed in different studies (Cabrera-Rubio et al., 2012; Chen et al., 2018).

Other factors less explored but described to affect the milk microbiome are summarized in Table 1 and include environmental exposure to disinfection agents (Bever et al., 2018), chemotherapy (Urbaniak et al., 2014b), maternal nutrient intakes (Boix-Amorós et al., 2016; Williams et al., 2017a), and the structure of human social networks, including cooperative breeding (Meehan et al., 2018). Correlation and network analyses have also related variation in the milk microbiome with variation in other milk components including immune cells, polyamines and fatty acids, though such observations have been reported in limited studies and their potential implications to monitor or improve maternal-infant health have not been thoroughly explored (Gómez-Gallego et al., 2017; Williams et al., 2017b). Despite of the large inter-individual and inter-population variations, most studies agree to identify the genera *Staphylococcus, Streptococcus*, and *Propionibacterium* as core members of the milk microbiota (Hunt et al., 2011; Jiménez et al., 2015), as they appear to be universally present in healthy milk irrespectively of maternal and infant characteristics studied to date. The presence of potentially beneficial bacteria including lactobacilli and bifidobacterial species, is also commonly reported in human milk, though these are not consistently detected across all samples and populations studied, and usually represent a minority of the bacterial populations detected (Chen et al., 2018).

Intriguingly, NGS-based studies have also identified in milk DNA from strictly anaerobic gut-associated microbes (*Bacteroides, Blautia, Clostridium, Collinsella, Coprococcus, Eubacterium, Faecalibacterium, Roseburia, Ruminococcus*,...), which are either non-culturable or hard to culture in the laboratory, and that have not been recovered through milk culturing to date (Cabrera-Rubio et al., 2012; Jost et al., 2013, 2014; Jiménez et al., 2015; Gomez-Gallego et al., 2016). Confirming their presence within healthy human milk would require its isolation through culturomic approaches or their detection through other techniques, including the specific labeling of target microorganisms (either through qPCR or flow-cytometry based approaches), or target specific RNA-based analysis, which would also confirm its functionality within the ecosystem and may provide additional proofs on the possible role of these bacteria for human health. Traditionally bacterial cells in milk were considered contaminants originating from the infant’s oral cavity or the mother’s skin. However, the detection of such anaerobic species typical from gut environments has suggested that breastfeeding could be an ingenious way to provide the newborn infant a set of commensal microbes to initially colonize the gut (McGuire and McGuire, 2015). Indeed, some works have proposed that selected bacteria in the maternal gut could reach the mammary gland through an endogenous route, involving complex interactions between bacteria, epithelial and immune cells (Martín et al., 2004). Although the mechanisms governing this process have not been elucidated yet, studies with pregnant and lactating mice models have offered a plausible scientific basis (Rodríguez, 2014; de Andrés et al., 2017; Mira and Rodriguez, 2017). Additionally, oral administration of some lactobacilli strains to lactating women led to their presence in milk supporting the existence of an endogenous gut-mammary gland connection during lactation (Abrahamsson et al., 2009; Arroyo et al., 2010).

Finally, metataxonomic studies have frequently revealed the presence in milk of sequences belonging to a group of soil- and water-associated bacterial genera, including *Acinetobacter, Bradyrhizobium, Methylobacterium, Microbacterium, Novosphingobium, Pseudomonas, Ralstonia, Sphingopyxis, Sphingobium, Sphingomonas, Stenotrophomonas*, and *Xanthomonas*, which have not been detected through milk culturing (Hunt et al., 2011; Cabrera-Rubio et al., 2012; Urbaniak et al., 2014a; Li S.-W. et al., 2017). In some works, the DNA sequences from such microorganisms were so frequent and abundant that were even consider part of the “core microbiome” of human milk (Hunt et al., 2011). However, contaminating DNA in PCR and DNA extraction reagents frequently belongs to these groups and may lead to artifacts when assessing low abundance microbiomes, such as human milk (Grahn et al., 2003; Mühl et al., 2010; Laurence et al., 2014; Salter et al., 2014; Kim et al., 2017; Perez-Muñoz et al., 2017). Thus, caution should be taken when analyzing the human milk microbiome using these methodologies.

## Past Pitfalls and New Opportunities

The constant refinement of culture-independent techniques has boosted our understanding of the milk microbial ecosystem although there are still limitations to unravel the study of this community. First, the analysis of low biomass microbiomes faces some challenges due to the risk of DNA contamination. Recommendations to reduce the impact of contaminants in sequence-based low-biomass microbiota studies have already been provided (Salter et al., 2014), including the utilization of DNA extractions procedures demonstrated to introduce the lowest DNA background possible. Besides, reporting sequencing of negative controls or describing identification and removal of contaminant sequences during the bioinformatics analysis of the sequencing reads (Davis et al., 2018; Karstens et al., 2018; Zinter et al., 2019), should be considered in milk microbiome studies.

Secondly, in view of the high inter-individual, inter-populations and inter-study variations reported to date, milk microbiome research needs an urgent standardization of samples collection and processing as previously proposed for other human microbiomes (Costea et al., 2017). Standardization should cover aseptic techniques, cautions to prevent sample contamination from skin or milk extraction devices, the convenience of using preservation protocols (Lackey et al., 2017); and DNA processing and sequencing analysis, which would enormously facilitate comparison of results from different studies (Costea et al., 2017). Besides, multiple maternal and environmental factors affect the milk microbiome, which together with the reduced number of samples analyzed in most available studies strongly hampers drawing biologically and universally valid significant conclusions. Defining appropriate inclusion/exclusion criteria and metadata collection is necessary to ensure the biological data obtained will answer the specific question under investigation. Furthermore, factors like maternal diet, known to affect the gut microbiota composition and some milk components, have not been considered in milk microbiomes studies and thus may need to be evaluated in further studies (Dingess et al., 2017; Bzikowska-Jura et al., 2018).

Most studies on milk microbiome to date have exclusively identified bacterial population shifts, but have not provided any functional insights into these communities. Results from gut microbiome studies have demonstrated that microbial ecosystems are more conserved at functional than at taxonomic levels, reflecting the existence of redundant functions among community members and suggesting that some species might be interchangeable without affecting the functional attributes of the community (Moya and Ferrer, 2016). Thus, functions may be better biomarkers for health-disease states than taxonomical composition though they have been scarcely studied on milk. A pooled milk sample resulting from 10 donors was analyzed through shotgun-sequencing, identifying abundant and prevalent functions in the milk microbiota, as compared to the infant gut microbiome and identified putative immunomodulatory motifs in microbiota-derived DNA sequences, providing basis for further mechanistic studies (Ward et al., 2013). Additionally, shotgun metagenomics enhance the resolution of taxonomic assignment and has demonstrated vertical transmission of strains from the mother to the infant (Asnicar et al., 2017); and identified novel functions of interest from unculturable bacteria in complex communities (Berini et al., 2017), though this potential has not been harnessed within the milk microbiota.

Although milk microbiome studies have focused on bacteria, milk may also vehiculate yeasts or viruses (Daudi et al., 2012; Dupont-Rouzeyrol et al., 2016; Mutschlechner et al., 2016). Since shotgun metagenomics sequences the whole DNA present at a given environment, including eukaryote, prokaryote and viral DNA, such approaches, together with 18S rRNAmetagenomic sequencing, have shed light on the existence of fungal species in milk samples from healthy women, opening new avenues to study other milk microbiota components that have been overlooked (Jiménez et al., 2015; Boix-Amorós et al., 2016). Similarly, a milk virome analyses recently revealed the dominance of bacteriophages as opposed to eukaryotic viruses (Pannaraj et al., 2018), raising the possibility of modulating milk bacterial populations by bacteriophages present within the community (Jiménez et al., 2015; Duranti et al., 2017).

It is worth highlighting that DNA-based studies provide information on the community’s composition and their metabolic potential but do not demonstrate the activities they perform *in situ*. To partly overcome such limitations, some studies have proposed the utilization of propidium monoazide (PMA), a DNA binding dye that enters only dead and membrane-compromised cells, thus preventing detection of damaged cells by qPCR or NGS approaches (Tantikachornkiat et al., 2016), although such strategies have been never reported on milk microbiota studies. Besides, a combination of flow-cytometry and NGS approaches has been proposed to specifically identify bacteria coated with host antibodies (Simón-Soro et al., 2015). In fact, bacterial interactions with the host immune system appear to differ between body sites and, understanding how the maternal immune system interacts with milk bacteria may help elucidate the mechanisms allowing bacterial translocation through an enteromammary pathway (Rodríguez, 2014). Other NGS-based methodologies that have not yet been applied to milk microbiota studies, include RNA-based (metatranscriptomics) or single-cell methodologies, which may offer novel information about the communities that are metabolically active at a given point and their functionality (Gosalbes et al., 2012; Yao et al., 2017).

**FIGURE 1:**
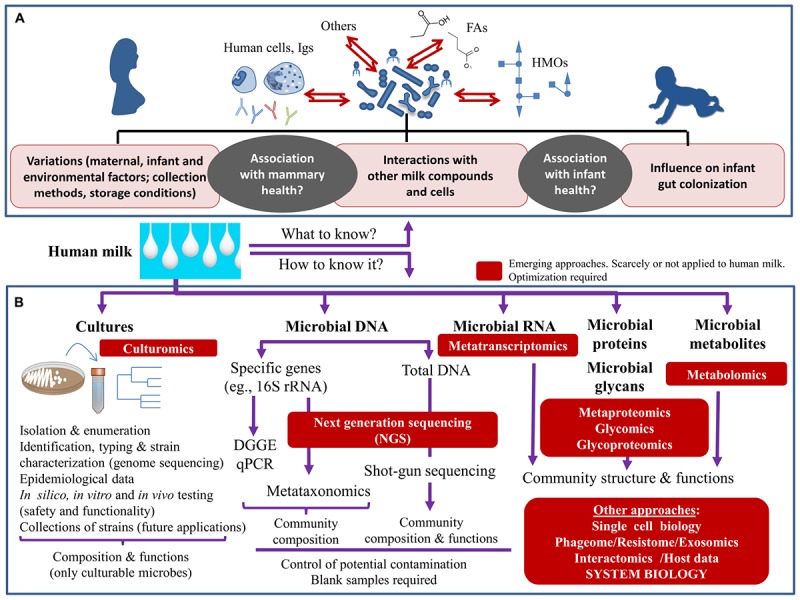
Schematic overview of what we need to know about the human milk microbiome **(A)** and the main tools that can be applied to achieve their study **(B)**.

Finally, little attention has been paid to the associations of milk microbiota with other milk components (Roncada et al., 2013; Kumar et al., 2016; Bardanzellu et al., 2017; Gómez-Gallego et al., 2017, 2018; Figure 1) although the combination of multiple “-omic” approaches, at DNA, RNA, protein and metabolite levels, will undoubtedly facilitate our comprehension on the inter-relationships among milk components, and ultimately, with the host. Besides, further expansion of culturing conditions, coupled to anaerobic maintenance of samples from collection to processing, will facilitate the isolation and recovery of microorganisms currently recalcitrant to grow under laboratory conditions (Stewart, 2012; Browne et al., 2016), enabling further mechanistic and functional studies. In conclusion, the constant refinement in culture-independent techniques has shed important information on the composition and modulation of the milk microbiome, although there are still important knowledge gaps on its functions and modulation. Emerging approaches offer novel opportunities to address studies in this field contributing to identify the mechanisms governing the milk microbiota assembly and its impact on maternal-infant health, paving the way to design novel microbiota-based strategies to promote maternal-infant health.

## Author Contributions

LR and JR conceived this review article. All authors wrote and approved the final version of the manuscript.

## Conflict of Interest Statement

CG-C is now employed by Probisearch SL, but did not at the time of the research being undertaken. The company had no role in the study. The remaining authors declare that the research was conducted in the absence of any commercial or financial relationships that could be construed as a potential conflict of interest.
